# Psychotropic Medication Adherence and Its Associated Factors Among Schizophrenia Patients: Exploring the Consistency of Adherence Scales

**DOI:** 10.7759/cureus.46118

**Published:** 2023-09-28

**Authors:** Maghzoub M Ali, Manal M Taha, Anas E Ahmed, Suhaila Ali, Maisa A Baiti, Atyaf A Alhazmi, Bushra A Alfaifi, Rania Q Majrabi, Nidaa Q Khormi, Alyaj A Hakami, Rafa A Alqaari, Raffan A Alhasani, Siddig I Abdelwahab

**Affiliations:** 1 Medical Research Center, Jazan University, Jazan, SAU; 2 College of Medicine, Jazan University, Jazan, SAU

**Keywords:** scales, internal consistency, adherence scales, schizophrenia, medication adherence

## Abstract

Background and objective

Non-adherence to psychotropic medication can aggravate an individual's illness, diminish treatment efficacy, or make patients less responsive to future therapeutic interventions. There are several scales available to measure non-adherence to medications. In this study, we aimed to measure psychotropic medication adherence and its associated factors among schizophrenic outpatients in Saudi Arabia.

Methodology

A cross-sectional study was conducted with a view to measuring psychotropic medication adherence and its associated factors. The Medication Adherence Rating Scale (MARS) and the Drug Attitude Inventory-10 (DAI-10) were translated into Arabic, and their internal consistency was measured. The adjusted odds ratios (AOR) were calculated using logistic regression in the IBM SPSS Statistics software version 23 (IBM Corp., Armonk, NY).

Results

Spearman's rho correlation indicated a negative association between DAI-10 and MARS scores (r = -0.579; p<0.05). The Arabic version of MARS was more reliable than DAI-10, as evidenced by Cronbach's alpha value. Of note, 60.20% (n = 59) of the sample demonstrated high adherence levels. The adherence level based on MARS scoring remained unaffected (p>0.05) in terms of gender, age, employment, marital status, educational level, income level, and duration of sickness. These results were obtained by using the multivariate logistic regression model; 89% of respondents reported not using psychiatric drugs given by someone else, despite the adherence rate not affecting this number.

Conclusion

The rate of non-adherence to psychotropic treatment was found to be high in our cohort. Hence, it is imperative to develop comprehensive intervention methods targeting the causes of non-adherence to psychiatric medication.

## Introduction

Psychiatric illnesses represent a significant global public health concern and have a prevalence of around 450 million individuals worldwide. Non-adherence to medication exacerbates the non-fatal disease burden associated with these conditions, which accounts for 30% of the worldwide burden of diseases. Additionally, it accounts for 14% of the entire global burden. In 2010, the economic burden of psychiatric disorders amounted to an estimated US$2.5 trillion, with projections indicating a substantial increase to US$6.0 trillion by the year 2030 [1]. Moreover, there are indirect financial costs that encompass several factors, such as lost resources and production, unemployment, absenteeism from work, and early mortality [1-2]. The World Health Organization (WHO) has established an all-encompassing strategic action plan (2013-2030) to advance mental well-being, avert psychiatric diseases, and deliver care and assistance to mitigate morbidity, disability, and death [2].

Approximately 31.7% of individuals who suffer from significant psychiatric problems experience prolonged impairment and dependence [3,4]. Psychiatric diseases are significantly linked to individual characteristics and community social support, cultural influences, social protection, living conditions, and various other environmental factors [2]. Ensuring medication adherence is critical in treating severe mental diseases [4-6]. According to WHO, medication non-adherence is defined as a situation wherein an individual's actions towards medicine intake do not align with the prescribed recommendations by healthcare professionals [7]. Individuals diagnosed with significant psychiatric disorders are prone to exhibiting non-adherence to prescribed drug regimens, mostly due to impaired cognitive thinking abilities and a limited understanding of their condition and corresponding treatment options. Factors linked to non-adherence in patients with schizophrenia include lack of insight, side effects, cognitive impairments, stigma, poor social support, weak therapeutic alliance, substance abuse, complex medication regimens, and financial constraints [4,8,9].

The Drug Attitude Inventory-10 (DAI-10) is widely recognized as the predominant self-report measure of compliance in the field of psychiatric research [10,11]. However, the DAI's validity might be questioned on several fronts. Primarily, according to research by Fenton et al. (1997), the validity of the scale is essentially dependent on the therapists' assessment, which has been shown to overstate compliance [12]. Secondly, the binary distinction between individuals who comply and those who do not comply often ignores the complexity of compliance, which is not a one-or-the-other phenomenon [12]. Third, the construct validity of this measure may be compromised since the DAI examines patient sentiments around their medicine rather than compliance behavior. Together, these issues cast doubt on the measure's efficacy because there is a lack of unbiased data to support its construct validity [13]. Thompson et al. (2000) conducted a study where they highlighted many limitations in the DAI as a tool for measuring adherence [14]. To address these, they created a new inventory called the Medication Adherence Rating Scale (MARS). MARS combines elements from both the DAI and the Morisky Adherence Scale (MAQ) developed by Morisky and colleagues [15]. Thompson et al. argued that MARS had enhanced validity and clinical value compared to the DAI. The researchers argued that the measure employed was both valid and reliable in assessing adherence to psychoactive drugs.

Non-adherence to psychotropic medication can result in worsening an individual's illness, diminished treatment efficacy, or decreased responsiveness to future therapeutic interventions. Additional ramifications of non-adherence include the need for readmission to medical facilities, reduced quality of life or psychosocial effects, recurrence of symptoms, heightened prevalence of comorbidities, inefficiency in allocating healthcare resources, and an elevated risk of suicide [3,4,15-18]. An analysis of the empirical data about the extent of non-adherence to psychotropic medication and the factors that contribute to it among individuals diagnosed with major mental illnesses is crucial for developing suitable interventions to attain desirable treatment outcomes for patients and healthcare practitioners. No primary studies addressing this matter have been undertaken so far to provide evidence-based guidance for policy-making. Therefore, conducting such studies on the extent and determinants of non-adherence to psychiatric medication would be valuable in guiding policymakers and program developers. In light of this, the primary objective of this analysis was to assess the prevalence of non-adherence to psychiatric medication and its associated factors. A comparison between DAI-10 and MARS was carried out as part of this investigation.

## Materials and methods

We employed a cross-sectional observational design for the present study. The study was conducted at the Amal Psychiatric Outpatient Clinic in Jazan Province, the Kingdom of Saudi Arabia (KSA). The clinic provides a wide range of essential services to patients from diverse backgrounds. The facility is a medical center catering to individuals of all ages, including adults and children. The present investigation was carried out over a period spanning from December 2022 to April 2023. The study included 98 schizophrenic patients and this sample size was determined based on several previous studies [13,19]. The study's inclusion criteria were as follows: individuals aged 18 or older receiving care at the Amal Psychiatric Outpatient Clinic. Individuals under the age of 18 years, those who had been recently admitted to a medical facility, and individuals who had undergone surgical procedures within the preceding four months were excluded from the study. Recent surgeries may affect study variables or outcomes. Surgical procedures, especially those with significant physiological changes or complications, may confound research objectives. Recovery after surgery was another factor considered. The exclusion criterion ensured the participants' physical and psychological stability.

The study involved outpatient clinic patients of both genders. The Diagnostic and Statistical Manual of Mental Disorders, Fifth Edition (DSM-V) criteria were used for the diagnosis of schizophrenia. The research assistant approached all the patients identified for their consent. The researchers gained ethical approval from the Ethical Committee for Scientific Research at the Substance Abuse Research Center, Jazan University, KSA. Furthermore, the questionnaire incorporated the principle of informed consent, and the patient's permission was duly obtained before the commencement of data collection. Before data collection, every patient was requested to read and provide their signature on a consent form. The dataset did not include any crucial participant information, such as personal names, identification numbers, and phone numbers. The respondents were interviewed using a structured questionnaire. The questionnaire was designed in Arabic for the convenience of data collectors. The data collection proforma was designed to collect information on potential factors related to non-compliance with medication among psychiatric patients. The questionnaire collected data about demographic characteristics such as sex, age, marital status, educational level, job, income, and the duration of the psychiatric illnesses.

The present study aimed to evaluate the attitudes and perspectives of individuals diagnosed with schizophrenia regarding the utilization of psychiatric medications and their corresponding experiences. DAI-10 was employed as an assessment tool to achieve this objective [10]. DAI-10 comprises 10 items: six that patients completely compliant with medication would answer as "True", and four they would rate as "False". For each question, the correct response received a score of plus one, while the erroneous response received a negative one. The sum of all the pluses and minuses was used to determine the final score. Positive total scores indicated compliance, whereas negative total scores indicated non-compliance. MARS was also used in this study [20]. The MARS questionnaire comprises 10 items intended to elicit patient complaints of non-adherence; this method has been proven effective in prior studies [13,21]. The overall score of MARS ranges from 0 to 10, with a higher number denoting superior adherence. Scores of 5 and above were classified as high, indicating a satisfactory level of treatment adherence, while scores below 5 were classified as low, indicating poor treatment adherence [22].

A professional translator and a clinical psychologist both reviewed the scales' Arabic translation. The alteration in medication's intended use was assessed via two questions. These inquiries sought to ascertain if the individual had previously consumed psychiatric drugs that were prescribed for another individual and whether the individual had ever distributed pills supplied to them by a medical practitioner to another individual. A pilot study involving 20 people was conducted to see if the questionnaire's phrasing was understandable and straightforward. Before data collection, each participant in this pilot project was asked to read and sign a consent form. The data from the pilot study was examined, although it was not used in the main trial. The reliability scores (Cronbach's alpha) for DAI-10 and MARS were obtained [23].

Data analysis

Data entry and analysis were conducted using IBM SPSS Statistics version 23 (IBM Corp., Armonk, NY). Means, standard deviations (SD), frequencies, and percentages were used to present the study variables. At a deeper level of data analysis, inferential statistical techniques were employed to examine differences and associations. The correlation between DAI-10 and MARS scores was examined using Spearman's rho. Multivariate logistic regression was also used to confirm the role of these factors in medication adherence. The odds ratios (ORs) were specified with a p-value and 95% confidence intervals. The goodness of fit of the data to the logistic regression was verified using the Hosmer-Lemeshow test. A p-value less than 0.05 was considered statistically significant.

## Results

The demographic data of the participants are presented in Table 1. The response rate was 100%. All patients were diagnosed using the DSM-V's basic procedures. The sample consisted primarily of males (54.2%), of which more than half were under the age of 50 years. The ages of the participants ranged from 22 to 85 years, with the average age being 48.2 ±12.43 years. Approximately 55% of the sample had an intermediate- or elementary-level education, whereas approximately 28% lacked basic literacy qualifications. About 65% of individuals fell in the middle-income bracket, as per the Saudi national categorization. Remarkably, a staggering 81% of individuals diagnosed with schizophrenia were unemployed. Of note, 72% of the participants in the study had received their diagnosis five years prior to the data collection.

**Table 1:** Demographic characteristics of the sample

Variables	N (%)
Gender	
Male	52 (54.2)
Female	44 (45.8)
Age group, years	
Less than 30	4 (4.1)
30 - 39	14 (14.3)
40 - 49	32 (32.7)
50 - 59	21 (21.4)
More than 60	14 (14.3)
Marital status	
Single	19 (19.4)
Married	79 (80.6)
Education	
Illiterate	28 (28.6)
Primary school	39 (39.8)
Middle school	15 (15.3)
High school	15 (15.3)
Income level	
Medium	61 (64.9)
Poor	33 (35.1)
Occupation	
Employed	17 (17.3)
Unemployed	81 (82.7)
Residence	
Rural	64 (66)
Urban	33 (34)
Duration of the disease, years	
Less than 5	13 (13.4)
5 - 10	35 (36.1)
10 - 15	35 (36.1)
15 - 20	9 (9.2)
More than 20	5 (5.1)

**Figure 1:**
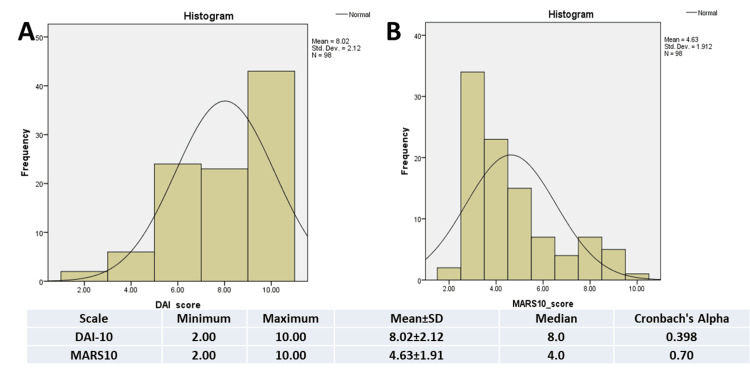
DAI-10 and MARS scores DAI-10: Drug Attitude Inventory-10; MARS: Medication Adherence Rating Scale; SD: standard deviation

The mean DAI-10 and MARS scores were 8.02 ±2.12 and 4.63 ±1.91, respectively. MARS was deemed more reliable than DAI-10, as evidenced by Cronbach's alpha coefficient (Figure 1). As shown in Figure 1, the DA1-10 score (Figure 1A) is skewed to the right, while MARS (Figure 1B) shows less left skewness. Spearman's rho correlation indicated a negative association between DAI-10 and MARS scores (r = -0.579; p<0.05).

**Figure 2:**
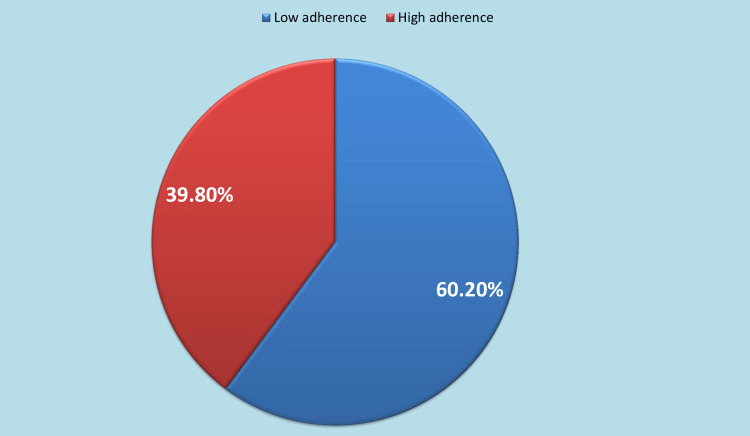
Adherence according to the MARS scoring system The overall score of MARS ranges from 0 to 10, with a higher number denoting superior adherence. Scores of 5 and above were classified as high, indicating a satisfactory level of treatment adherence, while scores below 5 were classified as low, indicating poor treatment adherence [22] MARS: Medication Adherence Rating Scale

The distribution of the participants based on the MARS scoring system is shown in Figure 2. Of note, 60.20% (n = 59) of the sample demonstrated high adherence.

Table 2 presents the data collected from the respondents regarding the alteration in the intended usage of psychiatric medications. Nine individuals, accounting for 9.2% of the sample, indicated that they had previously utilized psychiatric medications prescribed for someone else. Additionally, 15 individuals, representing 15.3% of the sample, reported sharing their prescription medications with others. These behaviors of altering the intended use of medications were not associated with the level of adherence as evidenced by the insignificant Chi-squared test results (Table 2).

**Table 2:** Alteration in the intended use of psychiatric medications *Chi-squared test

Items	Replies	MARS, n (%)		Test statistics* (p-value)
		Low adherence	High adherence	
Use of psychiatric drugs prescribed for someone else	Yes	4 (4.10%)	5 (5.1%)	1.03 (0.311)
	No	55 (56.1%)	34 (34.7%)	
Ever shared prescription drugs	Yes	8 (8.2%)	7 (7.1%)	0.35 (0.555)
	No	51 (52%)	32 (32.7%)	

The logistic regression model was employed to obtain the OR for all the analyzed factors (age, duration of the disease, education, income, marital status, residence, occupation, and gender). The dependent variable was medication adherence according to MARS. However, prior to that, it was essential to guarantee the validity of the study data to be involved in the model by using the Hosmer-Lemeshow test. The goodness of fit of our data to the multivariate logistic regression was ensured (p>0.05). None of the factors included in this study showed statistical significance (Table 3).

**Table 3:** Multivariate logistic regression

Variable	Categories	Odds ratio	95% confidence interval	
			Upper	Lower
Age group, years	Less than 30	0.00	0.00	0.00
	30 - 39	0.00	0.00	0.00
	40 - 49	0.60	0.09	4.20
	50 - 59	0.75	0.12	4.59
	More than 60 (ref)			
Duration of the disease, years	Less than 5 (ref)			
	5 - 10	2.68	0.18	40.31
	10 - 15	0.65	0.04	11.78
	15 - 20	1.75	0.06	49.45
	More than 20	2.09	0.05	82.57
Education	Illiterate (ref)			
	Primary school	0.99	0.17	5.84
	Middle school	0.99	0.07	13.73
	High school	2.39	0.21	27.77
Income level	Middle			
	Poor (ref)	0.30	0.07	1.36
Marital status	Single (ref)			
	Married	0.00	0.00	0.00
Residence	Rural (ref)			
	Urban	4.20	0.71	25.00
Occupation	Employed (ref)			
	Unemployed	1.95	0.23	16.68
Sex	Male (ref)			
	Female	2.34	0.40	13.60

## Discussion

The mean DAI-10 and MARS scores were 8.02 ±2.12 and 4.63 ±1.91, respectively. The Cronbach's alpha coefficient (Figure 1) showed that MARS is more dependable than DAI-10. The newly developed MARS shows promise of becoming a dependable and valid instrument for assessing medication compliance. Furthermore, this alternative method may possess a higher degree of validity when compared to the conventional DAI, which has historically been employed to assess medication adherence among individuals with mental conditions. One of the primary advantages of utilizing MARS is its inherent simplicity, facilitating its administration and interpretation in both clinical and research contexts. Compared to alternative approaches for measuring compliance, one notable feature of MARS is its comprehension of the nuanced nature of this behavior, as highlighted by Hughes et al. [13] (1997) and Kane [28] (1983). The utilization of this scale has been documented in this context. For instance, those who occasionally fail to adhere to their prescribed drug regimen are perceived as more compliant than those who intentionally choose to comply. Therefore, a score of 1 would suggest inadequate compliance, whereas 9 would imply higher levels of compliance.

The newly implemented compliance measure (MARS) effectively addresses certain challenges commonly encountered with other procedures. These alternative ways are often characterized by high costs in terms of both time and financial resources, without offering any significant improvement in terms of reliability [24-27]. The self-report measure may be readily implemented in many therapeutic settings, offering the advantages of efficiency and simplicity. This is attributed to its concise nature, consisting of 10 questions requiring a binary response (true or false). Clinicians may utilize this tool to enhance medication adherence among individual patients, as it assesses problematic behaviors through the MAQ questions and attitudes through the DAI-based items. Furthermore, it can be utilized in the broader context of study investigations that explore adherence and the determinants influencing adherence among psychiatric patients.

The findings of our investigation indicate that MARS exhibits significant reliability. It has exhibited a significant level of reliability in comparison to other self-report measures currently employed, and it has presented even more robust evidence than DAI. MARS possesses the added benefit of comprehending the intricacy of compliance behavior and provides a higher degree of value than several compliance measurement methods due to its versatility in various situations and its efficiency in terms of time and cost.

Prior comprehensive studies have demonstrated that medication non-adherence is a prevalent obstacle in the management of mental illnesses [28,29]. A comprehensive analysis of 16 studies involving 42,255 participants has revealed that the prevalence of medication non-adherence among individuals diagnosed with severe depressive disorders was estimated to be 50%, with a 95% confidence interval ranging from 40% to 59%. The prevalence observed in the sub-group study demonstrated a degree of consistency when compared to the total pooled prevalence, albeit slightly lower in the European region [30]. The mean for MARS is 4.63 ±1.91, which indicates weak adherence. The data in Figure 2 confirms this fact, where 60.20% of the sample is classified as low-adherence. These findings are consistent with previous studies from Pakistan [9], Nigeria [31], and Romania [32].

The MARS scores remained unaffected (p>0.05) by variables such as gender, age, employment, marital status, educational attainment, income level, and duration of sickness, which is in line with previous studies. The findings are based on the multivariate analysis. Ghosh et al. in their study in India found that adherence did not differ across the different categories of demographic variables [33,34]. Several factors may explain why our study did not find significant associations between medication adherence and age, gender, marital status, and educational level. These include differences in sample characteristics, variations in measurement methods, the multifactorial nature of medication adherence, and the possibility of chance or random variation. The composition of our sample differed from previous studies, thereby impacting the results. Our study's methods and measurement tools may have varied, affecting the observed relationships. Further research is necessary to comprehensively understand the complex relationship between medication adherence and these factors.

This study has a few limitations. The cross-sectional design of the study presented significant limitations that precluded the determination of causal relationships between our study findings and adherence within the sample population. Furthermore, it is essential to note that certain particular factors were not included in this study, potentially impacting the outcomes. The present research did not investigate the clinical variables linked to adherence to medication, including the severity of the disease, insufficient response to medication, or the level of awareness. The study's other limitations include the absence of assessment regarding individuals' attitudes toward medications, which could have influenced their adherence. Another limitation of this study is that it was confined to a single province in KSA, which means that its findings may not be generalizable to the broader Saudi population.

## Conclusions

This study is the first attempt to measure the level of concordance between ratings obtained from two self-administered Arabic language scales in the real-time setting of an outpatient psychiatric clinic. Based on our findings, MARS showed superior reliability and validity in assessing medication compliance. Furthermore, it is plausible that this measure is more valid than DAI, a conventional tool for assessing adherence to treatment among individuals with mental conditions. One notable advantage of MARS is its inherent simplicity, facilitating its hassle-free administration and interpretation in clinical and research contexts. The negative association between DAI-10 and MARS scores warrants further research.
